# *De Novo* Assembly of Venom Gland Transcriptome of *Tropidolaemus wagleri* (Temple Pit Viper, Malaysia) and Insights into the Origin of Its Major Toxin, Waglerin

**DOI:** 10.3390/toxins15090585

**Published:** 2023-09-21

**Authors:** Choo Hock Tan, Kae Yi Tan, Nget Hong Tan

**Affiliations:** 1 Department of Pharmacology, Faculty of Medicine, Universiti Malaya, Kuala Lumpur 50603, Malaysia; 2 College of Life Sciences and Medicine, National Tsing Hua University, Hsinchu 30013, Taiwan; 3 Department of Molecular Medicine, Faculty of Medicine, Universiti Malaya, Kuala Lumpur 50603, Malaysia; kytan_kae@um.edu.my (K.Y.T.); tanngethong@yahoo.com.sg (N.H.T.)

**Keywords:** transcriptomics, venomics, Wagler’s Pit Viper

## Abstract

The venom proteome of Temple Pit Viper (*Tropidolaemus wagleri*) is unique among pit vipers, characterized by a high abundance of a neurotoxic peptide, waglerin. To further explore the genetic diversity of its toxins, the present study *de novo* assembled the venom gland transcriptome of *T. wagleri* from west Malaysia. Among the 15 toxin gene families discovered, gene annotation and expression analysis reveal the dominating trend of bradykinin-potentiating peptide/angiotensin-converting enzyme inhibitor-C-type natriuretic peptide (BPP/ACEI-CNP, 76.19% of all-toxin transcription) in the transcriptome, followed by P-III snake venom metalloproteases (13.91%) and other toxins. The transcript TwBNP01 of BPP/ACEI-CNP represents a large precursor gene (209 amino acid residues) containing the coding region for waglerin (24 residues). TwBNP01 shows substantial sequence variations from the corresponding genes of its sister species, *Tropidolaemus subannulatus* of northern Philippines, and other viperid species which diversely code for proline-rich small peptides such as bradykinin-potentiating peptides (BPPs). The waglerin/waglerin-like peptides, BPPs and azemiopsin are proline-rich, evolving *de novo* from multiple highly diverged propeptide regions within the orthologous BPP/ACEI-CNP genes. Neofunctionalization of the peptides results in phylogenetic constraints consistent with a phenotypic dichotomy, where *Tropidolaemus* spp. and *Azemiops feae* convergently evolve a neurotoxic trait while vasoactive BPPs evolve only in other species.

## 1. Introduction

Snake venoms are evolutionary products that are structurally fine-tuned and functionally pre-validated by nature. The emergence of venom as a key innovation enables snakes to explore various ecological opportunities and adapt to new environments, leading to species diversification and speciation ultimately [1]. While occurring at varying rates, the diversification is accompanied by high evolvability of venom proteins subjected to positive selection and accelerated evolution among various lineages [2,3,4,5,6,7]. Most extant snakes deploy a single or a combination of major toxin families, which are, in a broad sense, predictable by the taxonomic lineage of a species. Nonetheless, venom phenotypes are not shaped by phylogenetic constraints *per se* but are likewise filtered by ecological interactions, permitting the existence of fitness optima and diversity through parallelism repeatedly attained by various species [8,9]. Hence, venom should be viewed as a key innovation, which itself evolves while providing snakes entry into new ecological niches [2,10]. As the ecological ranges expand and adaptive radiation becomes imminent, it is possible that some ecological conditions may pose constraints that favor evolutionary stasis and niche conservatism within certain lineages. Over macroevolutionary timescales, these factors reduce phenotypic variances of venom in a clade for individual fitness. Of particular interest is the genus of *Tropidolaemus*, a clade of Old World pit vipers that occupies a relatively basal position in the phylogeny of venomous snakes, and possesses a characteristic toxin arsenal uniquely streamlined to contain short neurotoxic polypeptides (waglerins) not found in other paraphyletic groups [11,12,13].

Widespread in the Indo-Malayan Archipelago, members of the *Tropidolaemus* genus (Family: Viperidae; Subfamily: Crotalinae) are regarded as potentially dangerous venomous snakes, although their medical significance in causing serious snakebite envenoming is vague [14]. The genus *Tropidolaemus* was first erected by Wagler in 1830 to accommodate a monotypic species [15], and soon synonymized as *Trimeresurus s. l.* (Asiatic lance-headed pit viper complex) (referred to *Trimeresurus subannulatus,* Gray, 1842) [16]. Based on morphological characteristics, *Tropidolaemus* was resurrected as a subgenus of the latter by Brattstrom [17], and later as a distinct genus by Burger [18]. This view was further supported by molecular phylogenetics [19,20,21], which identified *Tropidolaemus* as an ancient lineage of Old World pit vipers without close relationships to the various species within the *Trimeresurus* complex [13,22,23]. Together with *Deinagkistrodon acutus, Calloselasma rhodostoma*, *Garthius chaseni,* and *Hypnale* spp., they are regarded as basal pit vipers from Asia with somewhat species-poor genera known to date. Currently, four species of *Tropidolaemus* are established [13,24], including the most vastly described in literature and media, *Tropidolaemus wagleri* whose type locality was determined based on a neotype from the Sumatera Barat Province of Sumatra, Indonesia [25].

The species is known by the name Wagler’s Pit Viper, Speckled Pit Viper, and more commonly Temple Pit Viper owing to its famous association with the Temple of Azure Clouds (Snake Temple) in Penang Island, Malaysia. *Tropidolaemus wagleri* mainly distributes in the Indo-Malayan Archipelago, occurring in Southern Thailand and Southern Viet Nam, Peninsular Malaysia (including Penang Island and Pulau Pangkar), Singapore, and West Indonesia (Sumatra, Mentawei Archipelago, Natuna Islands, Nias, Riau Archipelago, and Bangka, but not Belitung) [13,25]. As with other *Tropidolaemus* species, the male and female snakes have similar external morphology in their juvenile phase while the adult snakes demonstrate remarkable sexual dimorphism, especially in length and girth where the female’s body size (up to about 100 cm in length) can be many times larger than a male’s (see graphical abstract in this work, and images in [12]). These snakes have remarkable morphological features including the absence of a nasal pore, upper surfaces of the snout, head covered with distinctly keeled small scales, and strongly keeled gular scales [13]. The heavily keeled feature is in contrast to the smooth scales seen in many other pit vipers, earning it the name of “Armored Viper” in some local languages.

*T. wagleri* is viviparous, and can be found perching on lower branches of trees in wet, low-elevation tropical rainforests [26]. As an arboreal ambush predator, this species uses venom to immobilize the prey and facilitate feeding. Owing to its venomous nature, *T. wagleri* is classified under Category 2 of medically important venomous snakes by the World Health Organization (WHO) [27]. Nonetheless, this species is generally docile, and its bite or envenoming rarely results in significant toxicity in humans [28]. Unlike the majority of Asiatic pit vipers, *T. wagleri* venom lacks hemorrhagic and coagulotoxic activities [29,30]. The venom is, however, lethal to mice with an intravenous median lethal dose of 0.5–0.6 µg/g, notwithstanding the sexual dimorphism of the snake [12]. The venom’s lethality is largely driven by waglerins, which are small basic polypeptides with a molecular mass of approximately 3 kDa [31,32,33]. The quantitative proteomic study of *T. wagleri* venom showed waglerins constitute ~17% of the total venom proteins; this is a unique venom phenotype not seen in other paraphyletic pit vipers [12,34]. Furthermore, the *T. wagleri* venom is antigenically distinct, lacking immunological recognition by antisera and antivenoms raised against other snake venoms [35,36]. Consistently, these biochemical, immunological, and proteomic studies suggest the uniqueness of *T. wagleri* venom properties. Beyond these, the transcriptomic profile of *T. wagleri* venom remains unexplored, restricting deeper insights into the diversity of toxin genes in this species. To remedy this, the present study assembled the venom gland transcriptome of *T. wagleri* from Malaysia through a *de novo* sequencing approach, followed by functional annotation and differential expression analysis of its toxin genes. The *de novo* transcriptome was further characterized by sequence analyses for the principal toxin, waglerin, to shed light on its evolutionary and medical implications.

## 2. Results and Discussion

### 2.1. De Novo Sequencing and Transcriptome Assembly

A total of 52,761,436 clean reads obtained through *de novo* sequencing were successfully assembled into 114,027 contigs with an N50 value of 546 (Table 1). Connecting these contigs yielded 58,914 transcripts (N50 = 1032), of which 54,781 with an expression level of FPKM > 1, were validated in the NCBI non-redundant (NR) protein database via BLASTx analysis (e-value < 10^−5^) (Supplementary File S1). These transcripts were further categorized into “unidentified”, “non-toxin”, and “toxin” categories accordingly. Gauged by FPKM, the toxin and non-toxin groups showed comparable expression levels, each constituting 40.07% and 37.59% of the total abundance of all transcripts, whereas the unidentified group has a lower expression level of 22.35%. Transcripts identified in the toxin group, however, are restricted to a small subset of 41 genes; in contrast, the non-toxin and unidentified groups contain thousands of transcripts, respectively. The high transcription of a relatively small number of toxin genes gave rise to an average expression level of 7840.78 FPKM per transcript in the toxin group in comparison to non-toxin and unidentified genes (93.33 and 88.69 FPKM/transcript, respectively), consistent with the primary function of the venom gland as a toxin-secreting organ. A similar trend is observed in a number of snake species whose toxin genes are highly co-expressed, suggesting genetic redundancy in support of venoms as integrated systems consisting of multiple paralogous toxins [37,38,39,40,41,42,43].

### 2.2. Toxin Gene Annotation and Expression Profile

**Figure 1:**
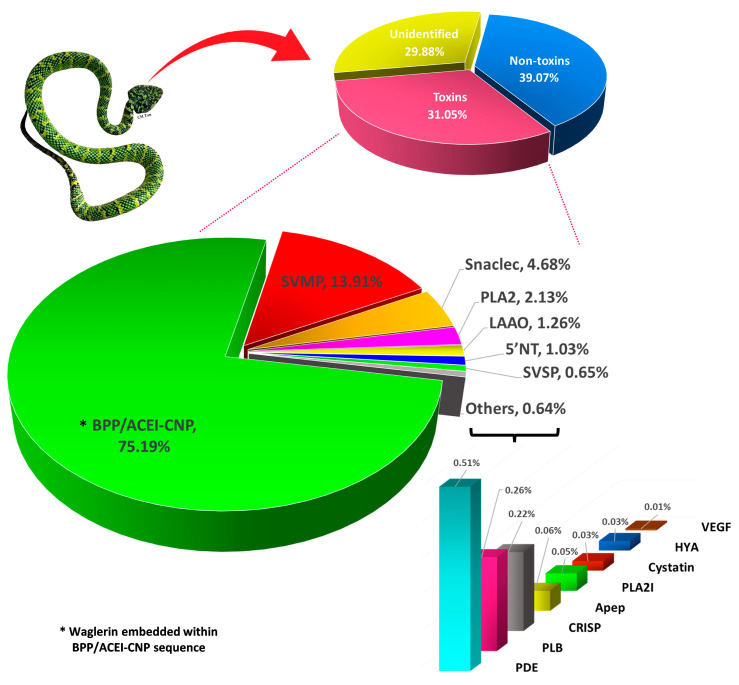
Overview of *Tropidolaemus wagleri* venom gland transcriptome. Upper panel: Classification of transcripts into toxins, non-toxins, and unidentified genes derived from *de novo* transcriptome assembly for the venom gland of Malaysian Temple Pit Viper (female) (inset). Lower panel: Profiling of toxin transcripts into 15 families of genes coding for various venom proteins. Percentages indicate the relative abundance of transcript based on FPKM. Abbreviations: BPP/ACEI-CNP, bradykinin-potentiating peptide/angiotensin-converting enzyme inhibitor-C-type natriuretic peptide; SVMP, snake venom metalloproteinase; Snaclecs, snake C-type lectins; PLA_2_, phospholipase A_2_; LAAO, L-amino acid oxidase; 5′NT, 5′-nucleotidase; SVSP, snake venom serine proteinase; PDE, phosphodiesterase; PLB, phospholipase-B; CRiSP, cysteine-rich secretory protein; Apep, aminopeptidase A; PLA2I, PLA_2_ inhibitor; HYA, Hyaluronidase; VEGF, vascular endothelial growth factor.

Transcripts within the toxin group were identified as 41 non-redundant genes with functional annotation based on various putative toxins of viperid snakes. These genes were compared using sequence homology and further assigned to 15 protein families (Table 2). The main transcript that dominates the overall transcription of toxins is functionally annotated to the precursor gene coding for a multi-domain protein, i.e., bradykinin-potentiating peptide/angiotensin-converting enzyme inhibitor-C-type natriuretic peptide (BPP/ACEI-CNP), constituting 75.19% of all-toxin FPKM. The second most abundant group of transcripts is snake venom metalloproteinase (SVMP, 13.91% of all-toxin FPKM), followed by snaclecs (SCL, 4.68%), phospholipase A_2_ (PLA_2_, 2.13%), L-amino acid oxidase (LAAO, 1.26%), and 5′-nucleotidase (5′NT, 1.03%). The remaining toxins have an expression level of <1% of all-toxin FPKM for each family; these are the snake venom serine proteinase (SVSP), phosphodiesterase (PDE), phospholipase B (PLB), cysteine-rich secretory protein (CRISP), phospholipase A_2_ inhibitor (PLA_2_ inhibitor), aminopeptidase, cystatin, hyaluronidase (HYA), dipeptidylpeptidase IV (DPP-IV), and vascular endothelial growth factor (VEGF) (Figure 1).

To note, the BPP/ACEI-CNP gene of *T. wagleri* embraces the coding region of waglerin, which is the most extensively studied venom protein originated from this species. Comparing the transcriptomic profile (current study) and reported proteome, the BPP/ACEI-CNP transcripts and waglerin proteins are both the most abundantly expressed [12,34]. This distinct venom characteristic is virtually exclusive to *T. wagleri*, representing a unique phenotype not typically evolved by other pit vipers. Other toxins expressed in the gland are derived from protein families shared across various lineages of hemotoxic pit vipers. These include enzymes such as proteases (SVMPs and SVSPs), phospholipases A_2_, L-amino acid oxidase, and non-enzymatic C-type lectins (snaclecs) and vascular endothelial growth factors. The amounts of transcripts transcribed and proteins translated, however, do not correlate exactly considering factors such as dynamic regulation of gene expression, differences in the synthesis rate and half-life of mRNA, and effect of post-translational modification [44,45]. The lack of correlation between venom gland transcriptomics and proteomics is not uncommon, as observed in various other species [37,44,46,47,48]. In these studies, the transcriptomic approach is used to reveal the gene expression pattern of all toxins at a single time point resembling a snapshot, typically a few days after snake venoms were extracted as an attempt to stimulate venom replenishment in the glands. On the other hand, venom proteomes show proteins that have been produced, matured, post-translationally modified, and accumulated in the glands over an unspecified duration. Nonetheless, the transcriptomic approach applying *de novo* assembly offers unmatched information on the expression patterns and structures (amino acid sequences) of species-specific toxins, with traceability to distinct genomic loci. This permits a better understanding of the evolutionary origin of venom, and how mutations could have modulated a venom’s phenotype in terms of biological function and toxicity. The insight is important for the characterization of a unique gene, as exemplified by the full-length BPP/ACEI-CNP gene of *T. wagleri* which codes for waglerin as the most abundantly expressed toxin.

### 2.3. Sequence Analysis and Characterization of Waglerin’s Precursor Gene

**Figure 2:**
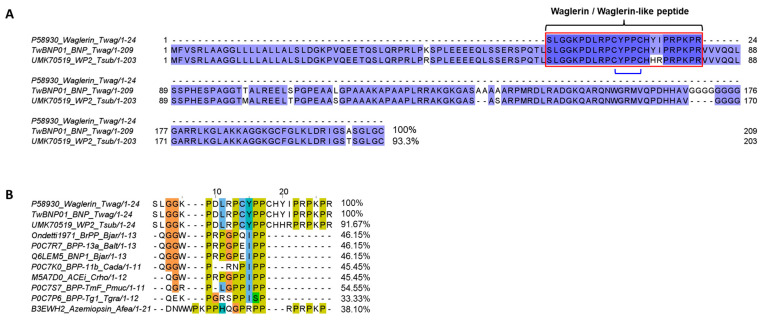
(**A**) Multiple sequence alignment of transcript TwBNP01 (current work, Malaysian *T. wagleri*) and homologous gene (Gene accession: UMK70519) (Northern Philippine *T. subannulatus*) with reference to the waglerin peptide (UniProt KB: P58930). Waglerin/waglerin-like peptide coding regions are indicated with a red box, and the blue semi-bracket indicates two conserved cysteine residues which form the disulfide bond. Color intensity indicates the identity of amino acid residues among the sequences. (**B**) Comparison of ten different proline-rich peptide sequences from multiple genera. Labeling of sequence follows a format of *Accession code_Protein name_Species*, except for “Ondetti1971” in panel B which is directly derived from the previous report [53]. Abbreviation of species names: *Twag*: *Tropidolaemus wagleri*; *Tsub*: *Tropidolaemus subannulatus*; *Bjar*: *Bothrops jararaca*; *Balt*: *Bothrops alternatus*; *Cada*: *Crotalus adamanteus*; *Crho: Calloselasma, rhodostoma*; *Pmuc*: *Protobothrops mucrosquamatus*; *Tgra*: *Trimeresurus gramineus*; *Afea*: *Azemiops feae*. Percentages next to all sequences indicate the degrees of amino acid sequence identity.

The transcriptome unveils the precursor gene of waglerin through the transcript TwBNP01, which contains a full-length sequence and has the highest expression level among all toxins expressed in the transcriptome (FPKM = 2,47,433; 75.19% of all-toxin FPKM) (Figure 1). BLASTp analysis matches TwBNP01 with 100% amino acid sequence coverage and 93.3% sequence identity (including additional or duplicated residues) to the gene UMK70519 (GenBank accession) found in the public database. The gene UMK70519 was derived from another species, *Tropidolaemus subannulatus* (locality: north Philippines, sequenced by Sangar’s method) [49], and deposited in the databank as “waglerin peptide 2” notwithstanding sequence variation from the original sequence of waglerin isolated from *T. wagleri* venom [50]. Both TwBNP01 and UMK70519 sequences are homologous to snake venom ACEI/BPP-CNP genes while exhibiting multiple mutations (mainly substitutions and deletions) accounted for sequence variation observed between the two (Figure 2A). The comparison suggests both TwBNP01 and UMK70519 are orthologous genes in *T. walgeri* (current study) and *T. subannulatus* [49], respectively.

*Tropidolaemus wagleri* and *T. subannulatus* are sister species diverged from a common ancestor and are both asympatric with non-overlapping biogeographic distribution [13]. Typically, *T. wagleri* distributes in the western part of the Indo-Malayan Archipelago whereas *T. subannulatus* are found eastward in islands such as Sulawesi and the Philippines. The venoms of the two congeneric species show remarkable variation in their reverse-phase chromatographic profiles in spite of close phylogenetic relationships [12]. While waglerin is highly expressed in the *T. wagleri* venom proteome (close to 20% of total venom proteins), a comprehensive venom proteome or protein isolation study is yet available to identify and quantify the corresponding orthologous protein in the *T. subannulatus* venom. Similarly, its expression level in a full transcriptomic study has yet to be reported for comparison with that of *T. wagleri*.

Waglerin was first purified from *T. wagleri* venom in the early 1990’s, and fully sequenced using the Edman degradation method (UniProt ID: P58930) [50,51]. In the present study, we identified the waglerin-coding region in the transcript sequence of TwBNP01 based on a 100% identity that matches the amino acid sequence of P58930: ^1^SLGGKPDLRPCYPPCHYIPRPKPR^24^. The 24 amino acid residues of waglerin were embedded in the propeptide region corresponding to the position from 58th to 82nd amino acid residues in the sequence of TwBNP01 transcript (Figure 2A). The waglerin sequence, however, shows variation from the corresponding region of UMK70519 in which two amino acid residues are non-synonymously substituted in *T. wagleri* and *T. subannulatus*: ^17^Y → ^17^H, ^18^I → ^18^R (Figure 2A). In both substitutions, the hydrophobic residues tyrosine and isoleucine (*T. wagleri*) are substituted for the positively charged histidine and arginine (*T. subannulatus*). Theoretically, the gene UMK70519 of *T. subannulatus* would produce a “waglerin-like” peptide with a higher peptide basicity than waglerin (P58930) and that of TwBNP01 in view of the substitutions. Indeed, computational predictions suggest the waglerin peptide (*T. wagleri*, current study) has a pI of 9.69 and a molecular mass of 2748.26 Da, while the waglerin-like peptide (*T. subannulatus*) has a pI of 10.31 and a molecular mass of 2765.26 Da. This has potential implications on the toxin activity, as a previous mutagenesis study showed the active site of waglerin is governed by basic residues that reside in the proximity of its disulfide linkage (shown in Figure 2A) [32,52]. Furthermore, it was suggested that the closer the basic amino acid residues are to the disulfide bond, the higher the waglerin toxicity would be [52]. Thus, the current finding implies waglerin from *T. wagleri* is relatively more conserved while substitutions occurring in UMK70519 (*T. subannulatus*) may result in enhanced toxicity of its waglerin-like peptide. This novel trait could be a key innovation for speciation that facilitates *T. subannulatus* in exploring and adapting to a new ecological niche as it diverged from *T. wagleri*. On this note, it is worth-mentioning that the waglerin-like peptide from *T. subannulatus* has yet to be characterized at the protein level to verify the postulated biological activity. Further studies are warranted for functional characterization and comparison of these peptides.

A BLAST search applying the two sequences (24 amino acid residues) yielded a minimal return of homology matches in the database, confirming waglerin and waglerin-like peptides are exclusively found only in the *Tropidolaemus* clade of Asiatic pit vipers. A previous study by Schmidt et al. [51] suggested similarity between waglerin and snake venom bradykinin-potentiating peptides (BPPs) by showing that waglerin and a BPP (from *Bothrops jararaca*) [53] are proline-rich with 50% homology. In comparison, the classical snake venom BPP has no disulfide bonds, contains fewer residues than waglerin, and has a blocked amino terminus (pyrrolidonecarboxylic acid as the amino-terminal residue). The current work took a closer look into the sequences of waglerin and various BPPs, and in addition, azemiopsin from the Fea’s Viper (*Azemiops feae*, subfamily: Azemiopinae) (Figure 2B). Alignment for homology inspection on these sequences shows the original waglerin (P58930) and the waglerin-coding sequence in TwBNP (current work) are indeed identical (100% identity) but varied from that of UMK70519 (*T. subannulatus*) (91.67% identity), and there is a considerably low degree of similarity between the *Tropidolaemus* peptides and those of BPPs of the other genera (~50% identity and below) including *Bothrops*, *Crotalus*, *Calloselasma*, *Protobothrops*, *Trimeresurus*, and *Azemiops*. Unlike the waglerin peptide, snake venom BPPs have no cysteine residues and, thus, the absence of disulfide bonds, contain fewer residues, and tend to have a blocked amino terminus where glutamine or glutamic acid forms pyrrolidonecarboxylic acid as the N-terminal residue (Figure 2B). These short peptides, nonetheless, are characterized by a high content of proline (P) residues which render them resistant to peptidase hydrolysis [54,55]. Notably, the “origins” of all these proline-rich peptides are embedded within bigger precursor genes of variable lengths recruited by different lineages in the Viperidae family.

**Figure 3:**
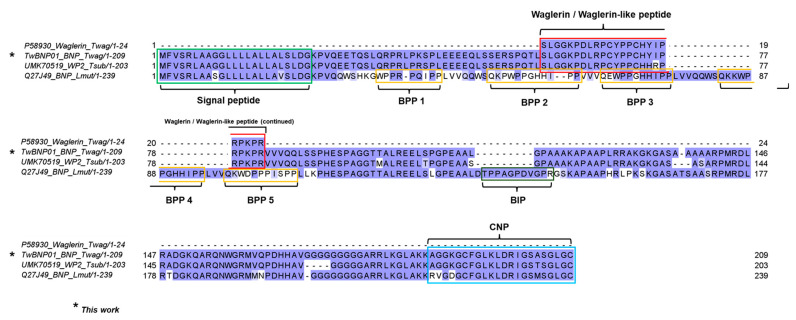
Multiple sequence alignment and annotation of protein-coding regions in the precursor gene of waglerin from *Tropidolaemus wagleri* (TwBNP01, current work) and *Tropidolaemus subannulatus* (UMK70519) with reference to the BPP/ACEI-CNP gene (Q27J49) from *Lachesis muta muta*. All entries share highly homologous signal peptides (light green box) and C-type natriuretic peptides (blue box). Waglerin/waglerin-like peptide-coding regions (red box) are present in *Tropidolaemus* spp. but not in *L. m. muta*, which instead evolves multiple bradykinin-potentiating peptides (BPPs) (orange boxes) and a bradykinin-inhibiting peptide (BIP) (dark green box). Color intensity of amino acid residues indicates the degrees of homology.

The protein-coding regions of precursor genes which share a homology of BPP/ACEI-CNP gene are hyper-variable, and have yet to be fully characterized for a number of species including the *Tropidolaemus* pit vipers. Thus, we further deduced the various putative protein-coding regions in TwBNP01 and UMK70519 sequences with reference to the well-characterized BPP/ACEI-CNP gene of *Lachesis muta muta* (UniProt ID: Q27J49) as the TwBNP01 sequence has a query coverage of 100% matched to Q27J49 (Figure 3). The analysis confirms that both sequences of *Tropidolaemus* species share a conserved structure resembling the BPP/ACEI-CNP gene of *L. m. muta*, containing a signal peptide sequence, a long propeptide sequence spanned by multiple short peptide-coding domains, followed by a long spacer gene with an intriguingly high content of repeated glycine (G) residue within a well-conserved poly-His-poly-Gly region toward their C-termini, where the CNP is coded for. In the precursor gene Q27J49 of *L. m. muta*, there are five domains coding for BPPs, one domain for bradykinin inhibitor peptide (BIP), and one CNP-coding domain [56,57]. Classically, BPPs are modular peptidic molecules with a C-terminal QIPP or HIPP and post-translationally modified N-terminal pyroglutamic acid (tryptophan in some variants) [55]. In comparison, these domains are not recognizable at all in both sequences of TwBNP01 (*T. wagleri*) and UMK70519 (*T. subannulatus*), suggesting *Lachesis* lineage either evolved the BPPs and BIP independently through mutations later at the genomic level, or the BPP/BIP sequences might have existed in the ancestral status but subsequently lost in the *Tropidolaemus* lineage. The first BPP (^34^WPPRPQIPP^42^) and second BPP (^50^QKPWPPGHHIPP^60^) sequences of *L. m. muta* show multiple substitutions from the corresponding segments in TwWAG01 and UMK70519, where the outcome significantly increases the content of proline residues while casting the C-terminal QIPP or HIPP motifs, which are characteristic of BPPs (Figure 3). Its third BPP domain (^65^QEWPPGHHIPP^75^) emerges more likely through duplication, while the fourth and fifth BPP are again the result of substitutions. The BPI domain (^137^TPPAGPDVGPR^147^) in Q27J49 is novel and present neither in TwWAG01 nor UMK70519 sequences. The CNP gene sequences, nonetheless, are conserved in the three species, in particular between TwWAG01 and UMK70519, where only one substitution (between Ala and Thr) occurred. The *L. m. muta* sequence is on the whole longer (239 amino acid residues) than the *Tropidolaemus sequences* (203–209 amino acid residues) (Figure 3), with suggestive features of duplication within its long propeptide and spacing gene regions that permit the evolution of molecular novelty.

### 2.4. Divergence and Conservation of BPP/ACEI-CNP Genes among Diverse Genera

**Figure 4:**
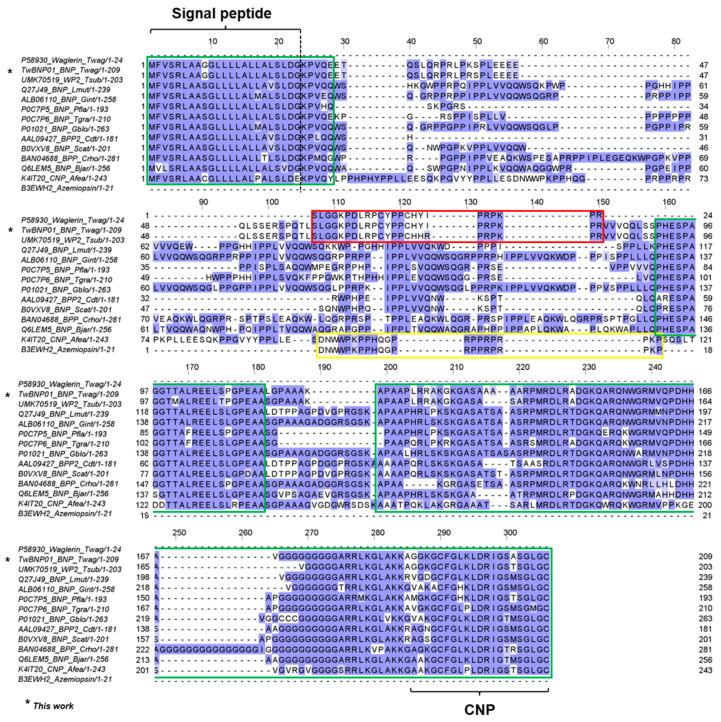
Multiple sequence alignment of homologous precursor genes encoding proline-rich peptides in various genera of pit vipers and Fea’s Viper. P58930 (waglerin) and B3EWH2 (azemiopsin) are short peptides included for comparison to indicate waglerin/waglerin-like peptide (red box) and azemiopsin (yellow box). Green boxes indicate regions with recognizable sequence similarity: (1) The signal peptide region and part of the initial propeptide (see overall residue numbering 1–28); (2) A long spacer gene involving the propeptide starting from PHESPAGGT- (residue numbering 159–181); (3) the CNP domain and its preceding propeptide (residue numbering 199–306). Identification of signal peptide and CNP domain was based on the BPP/ACEI-CNP gene of *Lachesis muta muta* (UniProt ID: Q27J49). Abbreviation of species names: *Twag*, *Tropidolaemus wagleri*; *Tsub*, *Tropidolaemus subannulatus*; *Lmut*, *Lachesis muta muta*; *Gint*, *Gloydius intermedius*; *Pfla*, *Protobothrops flavoviridis*; *Tgra*, *Trimeresurus gramineus*; *Gblo*, *Gloydius blomhoffii*; *Cdt*, *Crotalus durisus terrificus*; *Scat*, *Sistrusus catenatus*; *Crho*, *Calloselasma rhodostoma*; *Bjar*, *Bothrops jararaca*; *Afea*: *Azemiops feae*.

A wider comparison involving the transcript sequence of TwBNP01 and ACEi/BPP-CNP precursor genes from multiple genera of Crotalinae and *Azemiops feae* was illustrated in Figure 4. Three regions with considerable conservation were identified amidst the variable sequences: (1) The signal peptide region and part of the initial propeptide (see overall residue numbering 1–28); (2) A long spacer gene involving the propeptide starting from PHESPAGGT- (residue numbering 159–181) excluding the BIP region; (3) The CNP domain and its preceding propeptide (residue numbering 199–306). Across the sequences, the three conserved regions were bridged by highly variable amino acid residues of varying lengths. The variable segments form hypermutable regions within the propeptides, permitting the proline-rich peptides (waglerin, waglerin-like peptide, BPP, and azemiopsin) to evolve *de novo* from the large precursor genes [49], with neo-functionalization and adaption to new ecological niches and preys available for the different species.

Notwithstanding, the signal peptides are highly conserved among the genes compared. TwBNP01 and UMK70519 sequences have identical signal peptides (residues 1–23) which are, however, slightly variable from the rest of the species involving substitution of the 9th amino acid residue. Both *Tropidolaemus* spp. have ^9^Gly which was substituted for ^9^Ser in other species and ^9^Cys in *A. feae*. The signal peptide for *Tropidolaemus* can, therefore, be considered as genus-specific and potentially a useful genetic marker. A short stretch of amino acid residues of propeptide following the signal peptides shows similarity up to the 28th residue, where the ^28^Glu in *Tropidolaemus* was less conserved, with substitutions by Gln, Gly, and Tyr in the other species. The second conserved region containing residue ^159^PHESPAGGXTALREELSPGPEAA^181^ is of less predictive value for *Tropidolaemus* genes. Between TwBNP01 (*T. wagleri*) and UMK70519 (*T. subannulatus*), a substitution was found (^168^T → ^168^M, M appears to be a derived status), while this region in TwBNP01 is identical to that of *C. rhodostoma* (BAN04688). Interestingly, the gene K4IT20 from *A. feae* is variable due to its ambiguous sequence which suggests deletion of certain amino acid residues that follow the coding sequence for azemiopsin, preceding the supposedly conserved region. Lastly, the third conserved region comprising propeptides before the CNP domain is recognizable by the characteristic repeating poly-Ala and poly-Gly motifs across all species. The findings support the orthologous origin of the large precursor genes among these basal and advanced viperid snakes, while highlighting regions of vast variability that give rise to the emergence of diversely small, proline-rich bioactive peptides, notably waglerin, azemiopsin, BPP, and BIP in their venoms. The highly diverged sequences are evident in the propeptide regions (Figure 4), and this observation concurs with the hypothesis that “hyper-mutatable propeptide regions” are a source of molecular novelty in the course of the diversification of advanced snakes [49].

**Figure 5:**
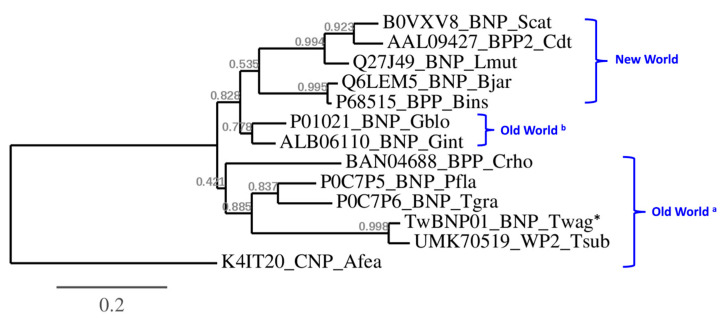
Phylogenetics of BPP/ACEI-CNP sequences of representative Viperidae species including TwBNP01 (*) obtained from the *de novo* assembled venom gland transcriptome of Malaysian *T. wagleri* (current work), and that coding for azemiopsin (K4IT20) of another subfamily (Azemiopsinae) as an outgroup. *T. wagleri* sequence is nested within the Old World paraphyletic clades of species mainly distributed in southeast Asia (indicated with superscript a), whereas the proteins of *Gloydius* species which have a further eastern dispersal in the Old World (indicated with superscript b) share a closer ancestral node with those of New World species. The waglerin precursor gene of *T. wagleri* (TwBNP01) and the gene coding for the waglerin-like peptide of *T. subannulatus* (UMK70519) are monophyletic, while the latter appears to be more derived. Branch support values are indicated in grey. The tree was reconstructed with maximum likelihood analysis with bootstrapping (100 replications), using MUSCLE (v3.7) for multiple alignments, Gblocks (v0.91b) for alignment curation, and PhyML (v3.0) for tree building. Graphical representation and edition of the tree were performed using TreeDyn (v198.3). Abbreviation of species names: *Twag*, *Tropidolaemus wagleri*; *Tsub*, *Tropidolaemus subannulatus*; *Afea*, *Azemiops feae*; *Tgra*, *Trimeresurus gramineus*; *Pfla*, *Protobothrops flavoviridis*; *Crho*, *Calloselasma rhodostoma*; *Gint*, *Gloydius intermedius*; *Gblo*, *Gloydius blomhoffii*; *Bins*, *Bothrops insularis*; *Bjar*, *Bothrops jararaca*; *Lmut*, *Lachesis muta muta*; *Cdt*, *Crotalus durisus terrificus*; *Scat*, *Sistrusus catenatus*.

Phylogenetic analysis of the BPP/ACEi-CNP precursor genes further revealed polyphyletic clades which conform to the biogeographical distribution of the species in comparison (Figure 5). The azemiopsin-containing precursor gene (K4IT20) is an outgroup to the rest of crotalid (pit viper) BPP/ACEi-CNP genes, consistent with its taxon as a separate subfamily (Azemiopinae). In the pit vipers (Crotalinae), two major paraphyletic clades are observed on the tree topology, namely the basal Old World group (represented by *Calloselasma*, *Trimeresurus*, *Protobothrops*, *Tropidolaemus*) and the New World group (*Bothrops*, *Lachesis*, *Crotalus*, *Sistrusus*), respectively, while the Asiatic *Gloydius* spp. appear closer to the latter (Figure 5). In the Old World clade, BAN04688 (equivalent UniProt: M5A7D0) from *C. rhodostoma* remains basal in the tree, and the ancestral protein further diversified along the speciation of *Trimeresurus*, *Protobothrops,* and *Tropidolamus* spp. (Figure 5). Of note, TwBNP01 (in which lies the waglerin peptide) originated from the Malaysian *T. wagleri* forms a monophyletic clade with the UMK70519 gene of *T. subannulatus* from North Philippines, with the latter sequence appears to be more diversified. The tree topology also shows an earlier diversification of BPP/ACEi-CNP proteins in the *Gloydius* spp. from members of their sister taxa in the Old World. From the common ancestor with *Gloydius*, the BPP/ACEI-CNP proteins diverged in the more derived New World species along with their speciation, and resulted in changes in the proteins’ physiological effects (Figure 5). From the perspective of phylogeography, the *Gloydius* spp. are regarded as the most eastward-dispersed Asiatic pit vipers, and are likely related to ancestral species which cross the Bering Bridge and radiate into the New World.

In Figure 4, multiple sequence alignment shows that waglerin/waglerin-like peptides and azemiopsin emerge within the highly diverged propeptide region, which otherwise gave rise to BPPs in the other species. The bradykinin-potentiating peptides (BPPs) and C-type natriuretic peptides (CNPs) of snake venoms exhibit vasoactive blood pressure-modulating activity, thus increasing the venom efficacy by subjecting the prey to a secondary subduing effect of the venom, e.g., a sudden reduction in blood pressure [55]. Although the precursor genes of TwBNP01 and UMK70519 contain the CNP regions, no expressed proteins have been reported from their venoms, while BPP-coding sequences simply never emerge *de novo* in both. We infer this as a phylogenetic constraint in which *T. wagleri* and *T. subannulatus* have no mechanistic way of evolving a venom phenotype that induces hypotension in prey. Instead, these pit vipers exclusively produce distinct peptides which functionally converge with alpha-neurotoxins (neurotoxic three-finger toxins) of the elapid species. In fact, waglerins are highly expressed as the principal toxins of *T. wagleri*, while in the other pit viper venoms, BPPs and CNPs are usually expressed at very low or negligible amounts, indicating secondary or ancillary functions [12,34]. The twist in the course of venom evolution taken by the *Tropidolaemus* species leads to the innovation of neurotoxic trait in this clade, where waglerin and waglerin-like peptides are neo-functionalized as the principal toxins to target and block the neuromuscular nicotinic receptors, producing paralysis as a rapid way of subduing the prey, especially in the arboreal habitat [33,58,59]. The use of small neurotoxic peptides is exemplified by another basal group of viperid snakes, i.e., *Azemiops* spp. (Fea’s vipers), from which the 21-amino acid residue peptide (also proline-rich) azemiopsin (UniProt ID: B3EWH2) is discovered [60]. Hence, within the Viperidae, the neurotoxic trait has been amplified on at least two separate occasions independently, once in the lineage of *Azemiops* and again in *Tropidolaemus* (Figure 5), notwithstanding their taxonomic divergence into two separate subfamilies (Crotalinae and Azemiopinae) and their distinct habitats (arboreal vs. ground-dwelling). The convergently evolved neurotoxic activities of waglerin and azemiopsin are taxon-specific though, resulting in differential effects in various animals where amphibians, avians, and rodents appear to be more susceptible than human [59]. Accordingly, these taxon-specific neuroactive peptides rarely result in significant neurotoxic syndrome in human envenoming cases. The molecules, nonetheless, may serve as lead compounds in drug discovery, cosmetic application (e.g., anti-wrinkle treatment inspired by waglerin), and receptor probes in biomedical research [61,62].

## 3. Conclusions

The present study *de novo* assembled the venom gland transcriptome of Temple Pit Viper, *T. wagleri* from west Malaysia. Gene annotation and expression analysis show that the BPP/ACEI-CNP transcript, TwBNP01, dominates the overall toxin transcriptions. The transcript represents a long precursor gene containing one waglerin-coding region, identified based on a sequence of 24 amino acid residues with 100% identity to the purified waglerin peptide known to date. The waglerin sequence is varied by substitutions of two amino acid residues from the waglerin-like peptide reported for its sister species, *T. subannulatus* of Northern Philippines. These peptides are exclusive to *Tropidolaemus* pit vipers, and are proline-rich as in bradykinin-potentiating peptides (BPPs) evolved by other viperid species as well as the azemiopsin peptide of Fea’s Viper, notwithstanding huge variations and a lack of homology in these short peptides among the different clades. The proline-rich peptides apparently evolve *de novo* from highly diverged propeptide regions within the orthologous genes of BPP/ACEI-CNP recruited by the various lineages, followed by neofunctionalization resulting in divergent activities of the peptides, notably characterized by a neurotoxic trait (waglerin and azemiopsin) and vasoactive (blood pressure-lowering) trait (bradykinin-potentiating peptides). While the genes coding for waglerin/waglerin-like peptides are nested within the paraphyletic Old World complex, there is no BPP-coding capacity shown by *T. wagleri* and *T. subannulatus*, as they diverge from the other pit viper species. Instead, the *Tropidolaemus* species and the Fea’s Viper convergently evolve small neurotoxic, nicotinic receptor-antagonistic peptides as their principal toxins, a unique venom phenotype emerges within the Viperidae family. Further research should delve into the differential activities of related peptides from a wider collection of various families for deeper insights into the evolution significance and bioprospecting potential of snake venom toxins.

## 4. Materials and Methods

### 4.1. Preparation of T. wagleri Venom Gland Tissue

The *Tropidolaemus wagleri* specimen was an adult female snake collected from Penang Island in the northwestern part of Peninsular Malaya. To promote transcription activity of the venom genes, venom milking was performed four days before the venom gland tissue was collected post-euthanasia [63]. The venom gland was sectioned into small pieces measuring 5 × 5 mm, and left overnight in RNAlater^®^ solution (Ambion, TX, USA) at 4 °C before transferring to −80 °C for storage. The protocol was approved by the Institutional Animal Use and Care Committee (IACUC) of Universiti Malaya, Malaysia (Approval code: #2013-11-12/PHAR/R/TCH).

### 4.2. RNA Extraction and mRNA Purification

The tissue was transferred into TRIzol solution (Invitrogen, Carlsbad, CA, USA) and homogenized in a 1 mL glass homogenizer. The total RNA was isolated with chloroform, and RNA-free DNAase I (Thermo Fisher Scientific, Waltham, MA, USA) was used to remove residual DNA. Purification of isolated RNA was achieved with the method of isopropyl alcohol precipitation. Oligo(dT) magnetic beads (Illumina TruSeq Stranded mRNA, San Diego, CA, USA) were used to extract the polyadenylated mRNA, and the quality of the total RNA was determined via the Agilent 2100 Bioanalyzer (RNA 6000 NanoKit, Agilent Technologies, Waldbronn, Germany).

The cDNA library was constructed using the poly(A)+ mRNA derived from the total RNA of the venom gland. Short fragments of the mRNA functioned as the templates for cDNA synthesis [64]. The first-stranded cDNA was synthesized using random hexamer-primer (N6), while the second-strand cDNA was synthesized using double-stranded cDNA. A paired-end library of the cDNA was formed using the Genomic Sample Prep kit (Illumina, San Diego, CA, USA). The cDNA fragments were purified using the QIAquick PCR extraction kit (Qiagen, Valencia, CA, USA), followed by end repair and addition of poly(A) to facilitate the ligation of Illumina adaptors which contain a single thymine (T) base overhang at their 3′ ends. Subsequently, polymerase chain reaction (PCR) was used to augment the cDNA fragments on a 1.5–2% TAE (Tris base, acetic acid and EDTA) agarose gel. Suitable fragments (200–700 bp) were identified as templates for subsequent PCR amplification. The amplified libraries were then sequenced in a single lane with 100-base-pair, paired-end reads using the Illumina HiSeq™ 2000 platform (Illumina, San Diego, CA, USA).

### 4.3. Filtration of Raw Reads

The sequenced reads were transformed via base calling into sequence data, identified as raw reads in a FASTQ format. The raw reads were filtered to produce clean reads before the assembly of transcriptome [65]. Reads with adaptors, those with >5% unknown nucleotides, as well as >20% low-quality bases were removed. Parameters for clean reads include Q20 percentage, defined as the proportion of nucleotides whose quality value is larger than 20 in reads; *N* percentage, which is the proportion of unknown nucleotides in reads; and GC percentage, the proportion of guanine and cytosine among all nucleotides.

### 4.4. De novo Assembly of Transcriptome

The *de novo* transcriptome assembly was achieved using a short-reads assembly program, Trinity (version 2.0.6) [66,67], where three independent software modules (Inchworm, Chrysalis, and Butterfly) were applied consecutively to process the sequenced reads (termed RNA-seqs). The process incorporated the algorithm of *de Bruijn* graphs construction, where k-mers (k = 25) were aligned and RNA-seqs with a certain length of overlap were linked to produce linear contigs. Contigs from the same transcript and the distance between them were determined, followed by clustering where each cluster has a complete set of *de Bruijn* graphs. The *de Bruijn* graphs, representing the transcriptional complexity at a given gene or locus, were independently processed to obtain transcripts for alternatively spliced isoforms and paralogous genes.

### 4.5. Functional Annotation of Transcripts

The transcript sequences were further processed for sequence splicing and redundancy removal using TGI clustering tools (TGICL, version 2.1) [68]. Consequently, non-redundant (NR) transcripts at the longest possible length were obtained and clustered into two classes of transcripts: (a) Clusters, labeled with a prefix of “CL” followed by the cluster ID as a contig; (b) Singletons, given a prefix of “Unigene”. Transcripts with sequence similarities >70% were gathered in one cluster, while those as singletons do not show sequence similarity at the given stringency.

All transcripts were then aligned with BLASTx to NCBI non-redundant protein database (NR), and a cut-off value of E < 10^−5^ was applied. The coding regions were determined based on BLASTx results, followed by translation into amino acid sequences using standard codon table. The length of the scaffold was extended based on overlapping sequences applying Phrap assembler (release 23.0) (http://www.phrap.org), and the longest non-redundant sequence in each cluster was selected as the transcript. For assembly success, the N50 length was set at N50 > 500 as an assembly quality indicator.

### 4.6. Quantifying Transcript Abundance

The transcripts were aligned using the Bowtie2 program [69], and their abundances were quantified by the RNA-seq using the expectation maximization (RSEM) tool [70]. For each transcript identified, its expression level was gauged using FPKM, fragments per kilobase of exon model per million reads mapped [71]. The FPKM normalizes read count based on gene length and the total number of mapped reads. The value was determined by using the RSEM tool based on a computational formula:

$$FPKM\;for\;gene\;A=\frac{10^{6}B}{NC/1000}$$

where FPKM indicates the expression of gene A; B refers to the number of reads aligned to the transcript of gene A; *N* is the total number of reads aligned to all transcripts; and *C* is the base number in the coding sequence of gene A.

### 4.7. Categorization of Transcripts

The *de novo* sequenced transcripts were subjected to BLASTx search to obtain the most resembling sequences available in the NR protein database. Transcripts with an FPKM value of more than 1 were recruited for categorization into three groups for the purpose of venom gene analysis; these are the “toxins,” “non-toxins”, and “unidentified” groups [37,39]. “Toxin” transcripts were searched for by applying snake toxin-related keywords as reported in previous transcriptomic works [37,39]. “Non-toxin” and “unidentified” groups contain transcripts of house-keeping genes and cellular proteins were assigned to the “non-toxin” group, while transcripts that could not be identified were classified accordingly as the “unidentified”. The average FPKM of each transcript in a group (total FPKM of respective group divided by the total number of transcripts in the group) indicates the redundancy value of gene expression [39]. The identities of transcripts in the toxin group were further validated by subjecting the amino acid sequences to the BLASTp suite (Basic Local Alignment Search Tool-Protein) analysis available in the NCBI and UniProt database platforms. The Serpentes database (taxid: 8570) was used in the search analysis, and the sequence identity was validated based on the E-score value and percentage of sequence similarity within the Viperidae family (vipers and pit vipers). The toxin transcripts were functionally categorized according to venom protein families based on proteins matched and abundances.

### 4.8. Sequence Alignment and Analysis

Multiple amino acid sequence alignment was performed with the aid of Jalview software (version 2.10.5; University of Dundee with Dundee Resource for Protein Sequence Analysis and Structure Prediction, Scotland, United Kingdom) [72] and MUSCLE (Multiple Sequence Comparison by Log-Expectation, version 3.8.31, for amino acids) [73] program. Sequences of related species used in sequence alignment analysis were retrieved from UniProtKB depository (accessed date: 5 April 2023) (http://www.uniprot.org/). The selection of sequences for use in the alignment analysis was based on their relevance to the toxin in comparison. The majority of sequences in alignment analysis were colored based on the BLOSUM62 color scheme, where the color intensity reflects the chance of amino acid substitution, i.e., intense purple = low chance of amino acid substitution; white = high chance of amino acid substitution. For comparison between TwBNP01 and UMK70519 sequences (Figure 2A), the coloration was based on sequence identity.

### 4.9. Phylogenetics

Sequences of TwBNP01 (current work) and related BPP/ACEI-CNPs were recruited to construct the phylogenetic tree. The tree was reconstructed using maximum likelihood analysis with bootstrapping (100 replications) in the advanced mode of the phylogeny.fr web server (http://www.phylogene.fr/ (accessed on 1 July 2023)) [74], using MUSCLE (v3.7) for multiple alignments, Gblocks (v0.91b) for alignment curation, and PhyML (v3.0) for tree building. Graphical representation and edition of the tree were performed using TreeDyn (v198.3).

### 4.10. Supporting Data

Sequencing data from the venom gland transcriptomics of *T. wagleri* was deposited in the National Centre for Biotechnology Information (NCBI) Sequence Read Archive (https://www.ncbi.nlm.nih.gov/sra/) (submitted on 21 July 2023) under SRA accession: PRJNA997256.

## Figures and Tables

**Table 1:** Overview of the NGS output statistics of *Tropidolaemus wagleri* venom gland transcriptomic analysis.

Parameter	Value
Total raw reads	56,736,590
Total clean reads	52,761,436
Total clean nucleotides (nt)	4,748,529,240
Q20 percentage	97.58%
*N* percentage	0.00%
GC percentage	49.68%
Contigs created	114,027
Total length (nt)	39,412,125
Mean length (nt)	346
N50	546
Transcripts assembled	58,914
Total length (nt)	39,179,883
Mean length (nt)	665
N50	1032
**Unigene/transcripts assembled (FPKM > 1)**	**54,781**
Unidentified	30,661
Non-toxin	24,079
Toxin	41

**Table 2:** Annotation and relative expression of toxin genes in the venom gland transcriptome of *Tropidolaemus waglari*.

Transcript Code	Annotated Protein Subtype	Matched Accession Code	Matched Species	Relative Abundance (%)
**Bradykinin-potentiating peptide/angiotensin-converting enzyme inhibitor-C-type natriuretic peptide (BPP/ACEI-CNP)**				**75.19%**
TwBNP01	Waglerin peptide 2	UMK70519	*Tropidolaemus subannulatus*	75.19%
**Snake venom metalloproteinase (SVMP): PIII class**				**13.91%**
Tw_SMP01	Zinc metalloproteinase-disintegrin-like VAP1	XP_039210133	*Crotalus tigris*	7.44%
Tw_SMP02	Zinc metalloproteinase-disintegrin HV1	Q90ZI3	*Protobothrops flavoviridis*	4.17%
Tw_SMP03	Zinc metalloproteinase-disintegrin-like 2d	J3SDW6	*Crotalus adamanteus*	2.30%
**Snaclec (SNL)**				**4.68%**
Tw_SCL01	Snaclec agglucetin subunit alpha-2	Q8AYA5	*Deinagkistrodon acutus*	2.97%
Tw_SCL02	Snaclec alboaggregin-D subunit beta	P0DM39	*Trimeresurus albolabris*	1.64%
Tw_SCL03	C-type lectin TsL	Q9YGP1	*Trimeresurus stejnegeri*	0.06%
**Phospholipase A_2_ (PLA_2_)**				**2.13%**
Tw_PLA01	Basic phospholipase A2 homolog acutohaemolysin	O57385	*Deinagkistrodon acutus*	1.47%
Tw_PLA02	Acidic phospholipase A2 Tgc-E6	A8E2V8	*Trimeresurus gracilis*	0.60%
Tw_PLA03	Basic phospholipase A2 Sms-N6	Q6EER6	*Sistrurus miliarius*	0.06%
Tw_PLA04	Basic phospholipase A2 Tbo-G6D49	Q2YHJ2	*Trmeresurus borneensis*	0.0005%
**L-amino acid oxidase (LAAO)**				**1.26%**
Tw_LAO01	L-amino acid oxidase Lm29	J7H670	*Lachesis muta*	1.26%
**5′nucleotidase (5′NT)**				**1.03%**
Tw_5NT01	Snake venom 5′-nucleotidase	F8S0Z7	*Crotalus adamanteus*	0.56%
Tw_5NT02	Snake venom 5′-nucleotidase	F8S0Z7	*Crotalus adamanteus*	0.47%
**Snake venom serine proteinase (SVSP)**				**0.65%**
Tw_SSP01	Venom plasminogen activator	E5L0E5	*Agkistrodon piscivorus leucostoma*	0.40%
Tw_SSP02	Thrombin-like enzyme gyroxin analog	P33589	*Lachesis muta muta*	0.14%
Tw_SSP03	Beta-fibrinogenase mucrofibrase-3	Q91509	*Protobothrops mucrosquamatus*	0.11%
Tw_SSP04	Beta-fibrinogenase mucrofibrase-3	Q91509	*Protobothrops mucrosquamatus*	0.09%
Tw_SSP05	Bradykinin-releasing enzyme KR-E-1	Q7SZE2	*Gloydius ussuriensis*	0.09%
Tw_SSP06	Thrombin-like enzyme stejnobin	Q8AY81	*Trimeresurus stejnegeri*	0.07%
Tw_SSP07	Thrombin-like enzyme acutobin	Q9I8X2	*Deinagkistrodon acutus*	0.07%
Tw_SSP08	Serine protease VLSP-3	E0Y420	*Macrovipera lebetina*	0.05%
Tw_SSP09	Thrombin-like enzyme KN-BJ 2	O13069	*Bothrops jararaca*	0.02%
**Phosphodiesterase (PDE)**				**0.51%**
Tw_PDE01	Venom phosphodiesterase 1	J3SEZ3	*Crotalus adamanteus*	0.33%
Tw_PDE02	Venom phosphodiesterase 1	J3SEZ3	*Crotalus adamanteus*	0.18%
**Phospholipase B (PLB)**				**0.26%**
Tw_PLB01	Phospholipase B	F8S101	*Crotalus adamanteus*	0.13%
Tw_PLB02	Phospholipase B	F8S101	*Crotalus adamanteus*	0.12%
**Cysteine-rich secretory protein (CRISP)**				**0.22%**
Tw_CRP01	cysteine-rich secretory protein Og-CRPa	Q7ZTA0	*Agkistrodon piscivorus piscivorus*	0.22%
**Phospholipase A_2_ inhibitor (PLA_2_ inhibitor)**				**0.05%**
Tw_PLI01	Phospholipase A2 inhibitor	O93233	*Gloydius brevicaudus siniticus*	0.05%
**Aminopeptidase**				**0.06%**
Tw_AP01	Aminopeptidase A	A5HUI5	Gloydius brevicaudus	0.06%
**Cystatin**				**0.03%**
Tw_CYS01	Cystatin-C	J3SE80	*Crotalus adamanteus*	0.02%
Tw_CYS02	Cystatin-1	J3RYX9	*Crotalus adamanteus*	0.01%
**Hyaluronidase (HYA)**				**0.03%**
Tw_HYA01	Hyaluronidase	J3S820	*Crotalus adamanteus*	0.03%
**Vascular endothelial growth factor (VEGF)**				**0.005**
Tw_VGF01	Snake venom vascular endothelial growth factor toxin	Q330K6	*Protobothrops mucrosquamatus*	<0.005
Tw_VGF02	Snake venom vascular endothelial growth factor toxin	Q90X24	*Bothrops insularis*	<0.005
Tw_VGF03	Vascular endothelial growth factor A	P67860	*Protobothrops flavoviridis*	<0.005
Tw_VGF04	Vascular endothelial growth factor A	P67860	*Protobothrops flavoviridis*	<0.005
Tw_VGF05	Vascular endothelial growth factor A	P67860	*Protobothrops flavoviridis*	<0.005
Tw_VGF06	Vascular endothelial growth factor A	P67860	*Protobothrops flavoviridis*	<0.005
Tw_VGF07	Vascular endothelial growth factor A	P67860	*Protobothrops flavoviridis*	<0.005
Tw_VGF08	Vascular endothelial growth factor A	C0K3N4	*Agkistrodon piscivorus piscivorus*	<0.005

## Data Availability

Sequencing data from the venom gland transcriptomics of *T. wagleri* is accessible through the NCBI Sequence Read Archive (https://www.ncbi.nlm.nih.gov/sra/), with SRA accession: PRJNA997256.

## Supplementary Materials

The following supporting information can be downloaded at: https://www.mdpi.com/article/10.3390/toxins15090585/s1, Supplementary File S1.

## References

[1] Arbuckle K. (2017). Evolutionary Context of Venom in Animals.

[2] Barua A., Mikheyev A.S. (2020). Toxin expression in snake venom evolves rapidly with constant shifts in evolutionary rates. Proc. Biol. Sci..

[3] Shibata H., Chijiwa T., Oda-Ueda N., Nakamura H., Yamaguchi K., Hattori S., Matsubara K., Matsuda Y., Yamashita A., Isomoto A. (2018). The habu genome reveals accelerated evolution of venom protein genes. Sci. Rep..

[4] Kini R.M. (2018). Accelerated evolution of toxin genes: Exonization and intronization in snake venom disintegrin/metalloprotease genes. Toxicon.

[5] Casewell N.R., Wagstaff S.C., Harrison R.A., Renjifo C., Wuster W. (2011). Domain loss facilitates accelerated evolution and neofunctionalization of duplicate snake venom metalloproteinase toxin genes. Mol. Biol. Evol..

[6] Ogawa T., Chijiwa T., Oda-Ueda N., Ohno M. (2005). Molecular diversity and accelerated evolution of C-type lectin-like proteins from snake venom. Toxicon.

[7] Deshimaru M., Ogawa T., Nakashima K., Nobuhisa I., Chijiwa T., Shimohigashi Y., Fukumaki Y., Niwa M., Yamashina I., Hattori S. (1996). Accelerated evolution of crotalinae snake venom gland serine proteases. FEBS Lett..

[8] Barua A., Mikheyev A.S. (2019). Many Options, Few Solutions: Over 60 My Snakes Converged on a Few Optimal Venom Formulations. Mol. Biol. Evol..

[9] Sunagar K., Fry B.G., Jackson T.N., Casewell N.R., Undheim E.A., Vidal N., Ali S.A., King G.F., Vasudevan K., Vasconcelos V. (2013). Molecular evolution of vertebrate neurotrophins: Co-option of the highly conserved nerve growth factor gene into the advanced snake venom arsenalf. PLoS ONE.

[10] Stroud J.T., Losos J.B. (2016). Ecological Opportunity and Adaptive Radiation. Annu. Rev. Ecol. Evol. Syst..

[11] Alencar L.R.V., Martins M., Greene H.W. (2018). Evolutionary History of Vipers. Encyclopedia of Life Sciences.

[12] Tan C.H., Tan K.Y., Yap M.K.K., Tan N.H. (2017). Venomics of *Tropidolaemus wagleri*, the sexually dimorphic temple pit viper: Unveiling a deeply conserved atypical toxin arsenal. Sci. Rep..

[13] Vogel G., David P., Lutz M., Van Rooijen J., Vidal N. (2007). Revision of the *Tropidolaemus wagleri* complex (Serpentes: Viperidae: Crotalinae). I. Definition of included taxa and redescription of *Tropidolaemus wagleri* (Boie, 1827). Zootaxa.

[14] Tan N.H., Tan K.Y., Tan C.H., Mackessy S.P. (2021). Snakebite in Southeast Asia: Envenomation and Clinical Management. Handbook of Venoms and Toxins of Reptiles.

[15] Wagler J.G. (1830). Natürliches System der Amphibien: Mit. vorangehender Classification der Säugethiere und Vögel: Ein Beitrag zur vergleichenden Zoologie.

[16] Gray J.E. (1842). Synopsis of the species of rattle-snakes, or family of Crotalidae. Zoological Miscellany 2.

[17] Brattstrom B.H. (1964). Evolution of the pit vipers. Trans. San. Diego Soc. Nat. Hist..

[18] Burger W.L. (1971). Genera of Pitvipers (Serpentes: Crotalidae).

[19] Vidal N., Lecointre G. (1998). Weighting and congruence: A case study based on three mitochondrial genes in pitvipers. Mol. Phylogenet Evol..

[20] Malhotra A., Thorpe R.S. (2000). A phylogeny of the trimeresurus group of pit vipers: New evidence from a mitochondrial gene tree. Mol. Phylogenet Evol..

[21] Parkinson C.L., Campbell J.A., Chippindale P.T., Schuett G.W., Höggren M., Douglas M.E., Greene H.W. (2002). Multigene phylogenetic analysis of pitvipers, with comments on their biogeography. Biology of the Vipers.

[22] Malhotra A., Thorpe R.S. (2004). A phylogeny of four mitochondrial gene regions suggests a revised taxonomy for Asian pitvipers (*Trimeresurus* and *Ovophis*). Mol. Phylogenet Evol..

[23] Creer S., Pook C.E., Malhotra A., Thorpe R.S. (2006). Optimal intron analyses in the *Trimeresurus* radiation of Asian pitvipers. Syst. Biol..

[24] Kuch U., Gumprecht A., Melaun C. (2007). A new species of Temple Pitviper (*Tropidolaemus* Wagler, 1830) from Sulawesi, Indonesia (Squamata: Viperidae: Crotalinae). Zootaxa.

[25] Uetz P., Freed P., Aguilar R., Reyes F., Hošek J. The Reptile Database. https://reptile-database.reptarium.cz/species?genus=Tropidolaemus&species=wagleri.

[26] Orlov N., Ananjeva N., Khalikov R., Schuett G.W., Hoggren M., Douglas M.E., Greene H.W. (2002). Natural history of pitvipers in Eastern and Southeastern Asia. Biology of the Vipers.

[27] World Health Organization (2016). Guidelines for the Management of Snakebites.

[28] Ismail A.K., Abd Hamid M.N.H., Ariff N.A., Frederic Ng V.E.R., Goh W.C., Abdul Samat N.S., Osman A.M.Z., Safferi R.S., Mohamed Ismail Z. (2023). Frequency, clinical characteristics and outcomes of *Tropidolaemus* species bite envenomations in Malaysia. PLoS Negl. Trop. Dis..

[29] Tan N.H., Tan C.S. (1989). The enzymatic activities and lethal toxins of *Trimeresurus wagleri* (speckled pit viper) venom. Toxicon.

[30] Minton S.A. (1968). Preliminary observations on the venom of Wagler’s pit viper (*Trimeresurus wagleri*). Toxicon.

[31] Tsai M.C., Hsieh W.H., Smith L.A., Lee C.Y. (1995). Effects of waglerin-I on neuromuscular transmission of mouse nerve-muscle preparations. Toxicon.

[32] Schmidt J.J., Weinstein S.A. (1995). Structure-function studies of waglerin I, a lethal peptide from the venom of Wagler’s pit viper, *Trimeresurus wagleri*. Toxicon.

[33] Lin W.W., Smith L.A., Lee C.Y. (1995). A study on the cause of death due to waglerin-I, a toxin from *Trimeresurus wagleri*. Toxicon.

[34] Tasoulis T., Isbister G.K. (2017). A review and database of snake venom proteomes. Toxins.

[35] Leong P.K., Tan C.H., Sim S.M., Fung S.Y., Sumana K., Sitprija V., Tan N.H. (2014). Cross neutralization of common Southeast Asian viperid venoms by a Thai polyvalent snake antivenom (Hemato Polyvalent Snake Antivenom). Acta Trop..

[36] Tan C.H., Tan N.H., Sim S.M., Fung S.Y., Gnanathasan C.A. (2012). Immunological properties of *Hypnale hypnale* (hump-nosed pit viper) venom: Antibody production with diagnostic and therapeutic potentials. Acta Trop..

[37] Chong H.P., Tan K.Y., Tan N.H., Tan C.H. (2019). Exploring the diversity and novelty of toxin genes in *Naja sumatrana*, the Equatorial spitting cobra from Malaysia through *de novo* venom-gland transcriptomics. Toxins.

[38] Palasuberniam P., Tan K.Y., Tan C.H. (2021). *De novo* venom gland transcriptomics of *Calliophis bivirgata flaviceps*: Uncovering the complexity of toxins from the Malayan blue coral snake. J. Venom. Anim. Toxins Incl. Trop. Dis..

[39] Tan C.H., Tan K.Y. (2021). *De Novo* venom-gland transcriptomics of spine-bellied sea snake (*Hydrophis curtus*) from Penang, Malaysia—Next-generation sequencing, functional annotation and toxinological correlation. Toxins.

[40] Hofmann E.P., Rautsaw R.M., Strickland J.L., Holding M.L., Hogan M.P., Mason A.J., Rokyta D.R., Parkinson C.L. (2018). Comparative venom-gland transcriptomics and venom proteomics of four Sidewinder Rattlesnake (*Crotalus cerastes*) lineages reveal little differential expression despite individual variation. Sci. Rep..

[41] Suryamohan K., Krishnankutty S.P., Guillory J., Jevit M., Schröder M.S., Wu M., Kuriakose B., Mathew O.K., Perumal R.C., Koludarov I. (2020). The Indian cobra reference genome and transcriptome enables comprehensive identification of venom toxins. Nat. Genet..

[42] Dashevsky D., Rokyta D., Frank N., Nouwens A., Fry B.G. (2021). Electric Blue: Molecular Evolution of Three-Finger Toxins in the Long-Glanded Coral Snake Species Calliophis bivirgatus. Toxins.

[43] Yee K.T., Macrander J., Vasieva O., Rojnuckarin P. (2023). Exploring Toxin Genes of Myanmar Russell’s Viper, *Daboia siamensis*, through *De Novo* Venom Gland Transcriptomics. Toxins.

[44] Margres M.J., McGivern J.J., Wray K.P., Seavy M., Calvin K., Rokyta D.R. (2014). Linking the transcriptome and proteome to characterize the venom of the eastern diamondback rattlesnake (*Crotalus adamanteus*). J. Proteom..

[45] Casewell N.R., Wagstaff S.C., Wüster W., Cook D.A.N., Bolton F.M.S., King S.I., Pla D., Sanz L., Calvete J.J., Harrison R.A. (2014). Medically important differences in snake venom composition are dictated by distinct postgenomic mechanisms. Proc. Natl. Acad. Sci. USA.

[46] Tan C.H., Tan K.Y., Fung S.Y., Tan N.H. (2015). Venom-gland transcriptome and venom proteome of the Malaysian king cobra (*Ophiophagus hannah*). BMC Genom..

[47] Tan C.H., Tan K.Y., Ng T.S., Tan N.H., Chong H.P. (2023). De Novo Venom Gland Transcriptome Assembly and Characterization for *Calloselasma rhodostoma* (Kuhl, 1824), the Malayan Pit Viper from Malaysia: Unravelling Toxin Gene Diversity in a Medically Important Basal Crotaline. Toxins.

[48] Tan K.Y., Tan C.H., Chanhome L., Tan N.H. (2017). Comparative venom gland transcriptomics of *Naja kaouthia* (monocled cobra) from Malaysia and Thailand: Elucidating geographical venom variation and insights into sequence novelty. PeerJ.

[49] Xie B., Dashevsky D., Rokyta D., Ghezellou P., Fathinia B., Shi Q., Richardson M.K., Fry B.G. (2022). Dynamic genetic differentiation drives the widespread structural and functional convergent evolution of snake venom proteinaceous toxins. BMC Biol..

[50] Weinstein S.A., Schmidt J.J., Bernheimer A.W., Smith L.A. (1991). Characterization and amino acid sequences of two lethal peptides isolated from venom of Wagler’s pit viper, Trimeresurus wagleri. Toxicon.

[51] Schmidt J.J., Weinstein S.A., Smith L.A. (1992). Molecular properties and structure-function relationships of lethal peptides from venom of Wagler’s pit viper, *Trimeresurus wagleri*. Toxicon.

[52] Hsiao Y.M., Chuang C.C., Chuang L.C., Yu H.M., Wang K.T., Chiou S.H., Wu S.H. (1996). Protein engineering of venom toxins by synthetic approach and NMR dynamic simulation: Status of basic amino acid residues in waglerin I. Biochem. Biophys. Res. Commun..

[53] Ondetti M.A., Williams N.J., Sabo E.F., Pluscec J., Weaver E.R., Kocy O. (1971). Angiotensin-converting enzyme inhibitors from the venom of *Bothrops jararaca*. Isolation, elucidation of structure, and synthesis. Biochemistry.

[54] Cheung H.S., Cushman D.W. (1973). Inhibition of homogeneous angiotensin-converting enzyme of rabbit lung by synthetic venom peptides of *Bothrops jararaca*. Biochim. Biophys. Acta.

[55] Pimenta D.C., Spencer P.J. (2021). Bradykinin-potentiating and related peptides from reptile venoms. Handbook of Venoms and Toxins of Reptiles.

[56] Graham R.L., Graham C., McClean S., Chen T., O’Rourke M., Hirst D., Theakston D., Shaw C. (2005). Identification and functional analysis of a novel bradykinin inhibitory peptide in the venoms of New World Crotalinae pit vipers. Biochem. Biophys. Res. Commun..

[57] Soares M.R., Oliveira-Carvalho A.L., Wermelinger L.S., Zingali R.B., Ho P.L., Junqueira-de-Azevedo I.L., Diniz M.R. (2005). Identification of novel bradykinin-potentiating peptides and C-type natriuretic peptide from *Lachesis muta* venom. Toxicon.

[58] Molles B.E., Rezai P., Kline E.F., McArdle J.J., Sine S.M., Taylor P. (2002). Identification of residues at the alpha and epsilon subunit interfaces mediating species selectivity of Waglerin-1 for nicotinic acetylcholine receptors. J. Biol. Chem..

[59] Harris R.J., Zdenek C.N., Debono J., Harrich D., Fry B.G. (2020). Evolutionary Interpretations of nicotinic acetylcholine receptor targeting venom effects by a clade of Asian Viperidae snakes. Neurotox. Res..

[60] Utkin Y.N., Weise C., Kasheverov I.E., Andreeva T.V., Kryukova E.V., Zhmak M.N., Starkov V.G., Hoang N.A., Bertrand D., Ramerstorfer J. (2012). Azemiopsin from *Azemiops feae* viper venom, a novel polypeptide ligand of nicotinic acetylcholine receptor. J. Biol. Chem..

[61] Debono J., Xie B., Violette A., Fourmy R., Jaeger M., Fry B.G. (2017). Viper Venom Botox: The molecular origin and evolution of the waglerin peptides used in anti-wrinkle skin cream. J. Mol. Evol..

[62] Mackessy P.S., Mackessy P.S. (2021). Reptile Venoms and Toxins: Unlimited Opportunities for Basic and Applied Research. Handbook of Venoms and Toxins of Reptiles.

[63] Rotenberg D., Bamberger E., Kochva E. (1971). Studies on ribonucleic acid synthesis in the venom glands of *Vipera palaestinae* (Ophidia, Reptilia). Biochem. J..

[64] Wery M., Descrimes M., Thermes C., Gautheret D., Morillon A. (2013). Zinc-mediated RNA fragmentation allows robust transcript reassembly upon whole transcriptome RNA-Seq. Methods.

[65] Conesa A., Madrigal P., Tarazona S., Gomez-Cabrero D., Cervera A., McPherson A., Szcześniak M.W., Gaffney D.J., Elo L.L., Zhang X. (2016). A survey of best practices for RNA-seq data analysis. Genome Biol..

[66] Grabherr M.G., Haas B.J., Yassour M., Levin J.Z., Thompson D.A., Amit I., Adiconis X., Fan L., Raychowdhury R., Zeng Q. (2011). Full-length transcriptome assembly from RNA-Seq data without a reference genome. Nat. Biotechnol..

[67] Haas B.J., Papanicolaou A., Yassour M., Grabherr M., Blood P.D., Bowden J., Couger M.B., Eccles D., Li B., Lieber M. (2013). De novo transcript sequence reconstruction from RNA-seq using the Trinity platform for reference generation and analysis. Nat. Protoc..

[68] Pertea G., Huang X., Liang F., Antonescu V., Sultana R., Karamycheva S., Lee Y., White J., Cheung F., Parvizi B. (2003). TIGR Gene Indices clustering tools (TGICL): A software system for fast clustering of large EST datasets. Bioinformatics.

[69] Langmead B., Salzberg S.L. (2012). Fast gapped-read alignment with Bowtie 2. Nat. Methods.

[70] Li B., Dewey C.N. (2011). RSEM: Accurate transcript quantification from RNA-Seq data with or without a reference genome. BMC Bioinform..

[71] Mortazavi A., Williams B.A., McCue K., Schaeffer L., Wold B. (2008). Mapping and quantifying mammalian transcriptomes by RNA-Seq. Nat. Methods.

[72] Waterhouse A.M., Procter J.B., Martin D.M.A., Clamp M., Barton G.J. (2009). Jalview Version 2—A multiple sequence alignment editor and analysis workbench. Bioinformatics.

[73] Edgar R.C. (2004). MUSCLE: Multiple sequence alignment with high accuracy and high throughput. Nucleic Acids Res..

[74] Dereeper A., Guignon V., Blanc G., Audic S., Buffet S., Chevenet F., Dufayard J.F., Guindon S., Lefort V., Lescot M. (2008). Phylogeny.fr: Robust phylogenetic analysis for the non-specialist. Nucleic Acids Res..

